# Effects of exenatide on cardiac function, perfusion, and energetics in type 2 diabetic patients with cardiomyopathy: a randomized controlled trial against insulin glargine

**DOI:** 10.1186/s12933-017-0549-z

**Published:** 2017-05-19

**Authors:** Weena J. Y. Chen, Michaela Diamant, Karin de Boer, Hendrik J. Harms, Lourens F. H. J. Robbers, Albert C. van Rossum, Mark H. H. Kramer, Adriaan A. Lammertsma, Paul Knaapen

**Affiliations:** 1 Diabetes Center/Department of Internal Medicine, VU University Medical Center, de Boelelaan 1117, 1081 HV Amsterdam, The Netherlands; 2 Department of Cardiology, VU University Medical Center, de Boelelaan 1117, 1081 HV Amsterdam, The Netherlands; 3 Department of Radiology and Nuclear Medicine, VU University Medical Center, de Boelelaan 1117, 1081 HV Amsterdam, The Netherlands

**Keywords:** Diabetes mellitus type 2, Exenatide, Cardiac function, Myocardial perfusion, Myocardial oxidative metabolism

## Abstract

**Background:**

Multiple bloodglucose-lowering agents have been linked to cardiovascular events. Preliminary studies showed improvement in left ventricular (LV) function during glucagon-like peptide-1 receptor agonist administration. Underlying mechanisms, however, are unclear. The purpose of this study was to investigate myocardial perfusion and oxidative metabolism in type 2 diabetic (T2DM) patients with LV systolic dysfunction as compared to healthy controls. Furthermore, effects of 26-weeks of exenatide versus insulin glargine administration on cardiac function, perfusion and oxidative metabolism in T2DM patients with LV dysfunction were explored.

**Methods and results:**

Twenty-six T2DM patients with LV systolic dysfunction (cardiac magnetic resonance (CMR) derived LV ejection fraction (LVEF) of 47 ± 13%) and 10 controls (LVEF of 59 ± 4%, P < 0.01 as compared to patients) were analyzed. Both myocardial perfusion during adenosine-induced hyperemia (P < 0.01), and coronary flow reserve (P < 0.01), measured by [^15^O]H_2_O positron emission tomography (PET), were impaired in T2DM patients as compared to healthy controls. Myocardial oxygen consumption and myocardial efficiency, measured using [^11^C]acetate PET and CMR derived stroke volume, were not different between the groups. Eleven patients in the exenatide group and 12 patients in the insulin glargine group completed the trial. Systemic metabolic control was improved after both treatments, although, no changes in cardiac function, perfusion and metabolism were seen after exenatide or insulin glargine.

**Conclusions:**

T2DM patients with LV systolic dysfunction did not have altered myocardial efficiency as compared to healthy controls. Exenatide or insulin glargine had no effects on cardiac function, perfusion or oxidative metabolism.

*Trial registration* NCT00766857

## Background

Type 2 diabetes mellitus (T2DM) is associated with an increased risk of heart failure (HF), even after adjusting for coronary artery disease (CAD) and hypertension [1]. The optimal bloodglucose-lowering therapy in T2DM patients and HF is still under debate. Multiple agents [2–4] have been linked to cardiovascular events. Other agents [5, 6] have been associated with a lower risk of cardiovascular events.

Preliminary studies have shown recovery of left ventricular (LV) function during glucagon-like peptide-1 (GLP-1) administration in HF patients irrespective of the diabetic status [7, 8]. However, underlying mechanisms for this effect remain to be elucidated. Small scaled studies suggested enhanced endothelial function and increased perfusion by GLP-1 [9, 10]. Moreover, shift towards augmented glucose metabolism has favorable effects on cardiac energetics [11]. Both phenomena have important prognostic relevance [12, 13]. At present, effects of GLP-1 receptor agonists (RA) on myocardial perfusion and energetics in T2DM patients with LV systolic dysfunction are unclear. In view of the diabetes pandemic and the associated high risk of HF, bloodglucose-lowering agents that can be prescribed safely are of great importance. Therefore, the aim of this study was to examine effects of GLP-1RA on cardiac function, myocardial perfusion, and energetics in T2DM patients with LV systolic dysfunction compared to insulin glargine.

## Methods

### Participants

T2DM patients with LV dysfunction, LV ejection fraction (LVEF) < 50% [as documented in the medical records, measured using echocardiogram, radionuclide angiogram or cardiovascular magnetic resonance imaging (CMR)], above 18 years, body mass index (BMI) of 25–40 kg m^−2^, hemoglobin A1C (HbA1c) of 6.5–10.0% (48–86 mmol mol^−1^), were randomized, at an allocation ratio of 1:1, open-label, to exenatide or insulin glargine on top of ongoing use of oral glucose-lowering agents (metformin or metformin and sulfonylurea) after a run-in period of 10 weeks. Exclusion criteria were renal or liver impairment, malignancy, cardiovascular events <3 months, insulin, thiazolidinediones, incretin-based therapies <4 months and chronic glucocorticoid use. Patients with contraindication for positron emission tomography (PET) or CMR (e.g. claustrophobia, implanted metal devices, rhythm other than sinus) were excluded. Healthy BMI-matched subjects with normal glucose metabolism (assessed by 75-g oral glucose-tolerance test) served as controls. All participants gave written informed consent. The study protocol was approved by the Medical Ethics Review committee of the VU University Medical Center, and was performed in full compliance with the declaration of Helsinki.

### Study procedures

5 µg exenatide twice daily was injected subcutaneously, 15 min before breakfast and dinner, for 4 weeks, followed by an increase to 10 µg twice daily. Insulin glargine was initiated at 10 IU once daily, injected subcutaneously according to normal standard dosages. Patients were instructed to increase the dose based on fasting blood glucose levels (<5.6 mmol L^−1^) according to a prespecified treat-to-target algorithm [14]. CMR and PET were performed in the morning within 4 h after a standardized breakfast and study medication, prior to randomization, and patients underwent follow-up measurements after 26 weeks of treatment.

### Outcomes

The primary outcome in the current study was effects on LVEF after 26-week treatment of exenatide versus insulin glargine in T2DM patients with LV systolic dysfunction. Secondary outcomes were differences in myocardial perfusion and energetics in T2DM patients with LV systolic dysfunction versus healthy BMI-matched healthy controls, and effects on myocardial perfusion and energetics after 26-week treatment of exenatide versus insulin glargine in T2DM patients with LV systolic dysfunction.

### CMR

Measurements were performed using a 1.5 Tesla whole-body magnetic resonance imaging (MRI) scanner (MAGNETOM Avanto, Siemens, Erlangen, Germany). All images were acquired with electrocardiographic triggering, during repeated expiration breath-holds. Cine imaging was used to measure LV dimensions and systolic function. After localizing scout scans, cine images were acquired using a retrospectively triggered, balanced steady-state free precession (SSFP) gradient echo sequence in three long axis views (2-, 3-, and 4-chamber views). Subsequently, short axis images (slice thickness 5 mm, gap 5 mm), covering the whole LV from mitral valve annulus to apex, was acquired. Delayed contrast enhancement (DCE) imaging was used to quantify myocardial scarring. DCE images were acquired 15–20 min after intravenous administration of 0.2 mmol kg^−1^ gadolinium-based contrast agent (gadoterate meglumine, Dotarem^®^, Guerbet, France). DCE images were acquired in the same short and long axis views as those used for cine imaging. For this purpose, a 2D segmented inversion-recovery prepared gradient echocardiography sequence was applied.

### CMR data analysis

LVEF and global LV parameters were quantified using MASS software package (MEDIS, Leiden, The Netherlands). Endocardial and epicardial borders were outlined manually in end-diastolic and end-systolic frames of all short-axis slices. Presence, and degree of fibrosis, of total ventricular mass, were quantified from DCE images using the 5-standard deviation method [15].

### PET

PET assessments were performed using a hybrid PET/computed tomography (CT) scanner (Gemini TF-64, Philips Healthcare, Best, The Netherlands). Oxidative metabolism was measured using [^11^C]acetate and perfusion using [^15^O]H_2_O.

#### [^11^C]acetate PET scan

After a survey scan to position the patient, 370 MBq of [^11^C]acetate was injected intravenously (5 mL bolus, infusion speed 0.8 mL s^−1^) followed by a saline flush (35 mL, infusion speed 2 mL s^−1^). Simultaneously, an emission scan was started, acquiring list mode data for 50 min. Using a low-dose respiration-averaged CT scan (55 mA, rotation time 1.5 s, pitch 0.825, collimation 64 × 0.625, acquiring 20 cm in 11 s) tissue density was measured and [^11^C]acetate scan was corrected for tissue attenuation. Dynamic images were reconstructed using 3D-row action maximum likelihood algorithm into 36 frames (1 × 10, 8 × 5, 4 × 10, 3 × 20, 5 × 30, 5 × 60, 4 × 150, 6 × 300 s), applying all appropriate corrections.

#### [^15^O]H_2_O PET scan

Hyperemic scan was performed during adenosine intravenously at a rate of 140 mcg kg^−1^ min^−1^. Rest and hyperemic scans were acquired in a list mode of 6 min. At the start of each perfusion scan, 370 MBq [^15^O]H_2_O was injected intravenously (5 mL bolus, infusion speed 0.8 mL s^−1^) followed by a saline flush (35 mL, infusion speed 2 mL s^−1^). Both scans were followed by a low-dose respiration-averaged CT scan (55 mA, rotation time 1.5 s, pitch 0.825, collimation 64 × 0.625, acquiring 20 cm in 11 s). Images were reconstructed using 3D-row action maximum likelihood algorithm into 22 frames (1 × 10, 8 × 5, 4 × 10, 2 × 15, 3 × 20, 2 × 30, and 2 × 60 s), with application of all appropriate corrections. BP was measured every 3 min during the entire scan. An interval of at least 10 min was used between the end of the first and the start of the second [^15^O]H_2_O scan to allow for decay of radioactivity to background levels.

### PET data analysis

Regions of interest were drawn on resliced short axis projections of a static [^11^C]acetate image during maximum myocardial uptake according to the standardized AHA 17-segment model [16]. To obtain arterial input function, additional regions were drawn within the ascending aorta in at least five consecutive planes. Subsequently, all regions were projected onto all frames of the dynamic [^11^C]acetate scan to generate time-activity curves (TAC). A single-tissue compartment model with model based corrections for spillover, partial volume and recirculating [^11^C] activity was used to derive k_2_, representing the rate constant for tracer washout from myocardial tissue, described previously [17, 18]. Quantitative parametric myocardial blood flow (MBF) images were generated using software developed in-house [19]. MBF was expressed in mL min^−1^ g^−1^ of perfusable tissue.

### Data analysis

Rate pressure product (RPP) was obtained by multiplying systolic BP (SBP) with heart rate (HR). Resting MBF corrected for RPP was derived as MBF at rest divided by RPP at rest, multiplied by 10^4^. Coronary flow reserve (CFR) was defined as the ratio between hyperemic and resting MBF and CFR corrected for RPP as the ratio between hyperemic and resting MBF corrected for RPP. External work was defined as the product of mean arterial pressure and CMR derived stroke volume, converted to units of energy (Joule). The caloric equivalent of 1 mL oxygen ≈20 Joule, whereas 1 mm Hg mL equals 1.33∙10^−4^ Joule [20]. Myocardial oxygen consumption (MVO_2_) in mL g^−1^ min^−1^ was derived from k_2_ using estimated MVO_2_ = 1.35∙k_2_ − 0.0096 as described previously [21]. Myocardial efficiency was calculated as (External work·HR)/(MVO_2_·LV mass·20) [22].

### Statistical analysis

Data are expressed as means ± standard deviations (for normally distributed data) or medians (interquartile range). *T* test or Mann–Whitney U-test was used to determine within-group differences. Between-group comparisons were performed using linear regression analysis with adjustments for intervention group and baseline values. Correlation coefficients were calculated using Pearson’s correlation. Statistical analyses were performed using SPSS software version 20.0 (IBM corporation, New York, US). P-value <0.05 was considered as statistically significant. Sample size calculations are based on the primary efficacy endpoint, i.e. LVEF. According to previous studies using GLP-1 and based on clinical experience, an absolute LVEF improvement of 5% is regarded as attainable and clinically relevant. In order to detect an absolute 5% improvement of LVEF from baseline in patients treated with exenatide, relative to those treated by the comparator insulin glargine (SD absolute 4%; α = 0.05, β = 0.10), 13 patients in each group will be needed.

## Results

### T2DM patients versus controls

**Fig. 1:**
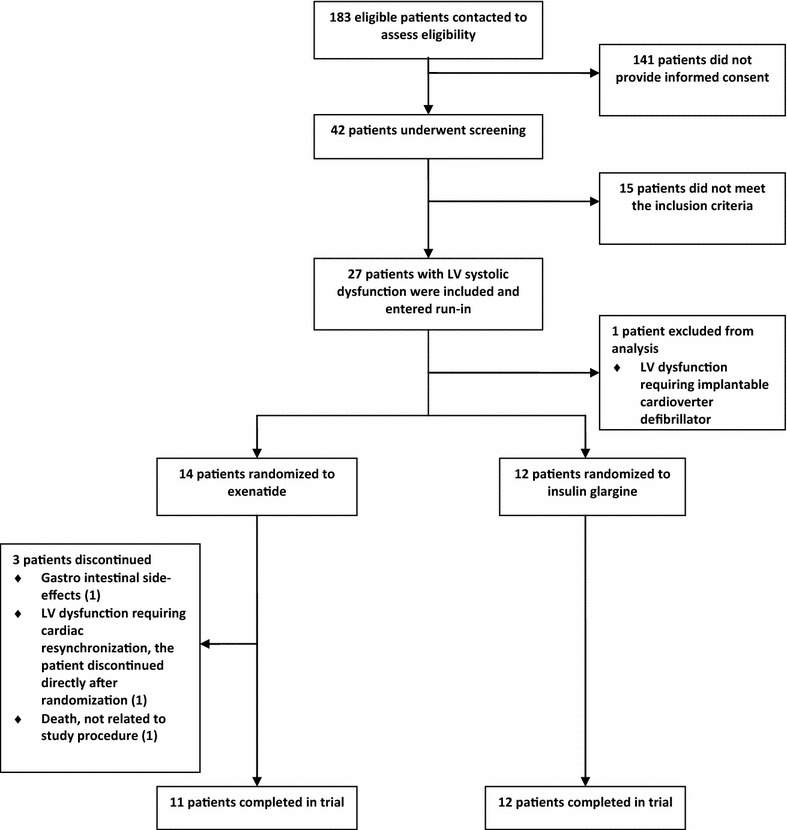
Flowchart of type 2 diabetic patient disposition. *LV* left ventricular

Twenty-seven T2DM male patients (Fig. 1) and 10 male controls were included. One patient was excluded from analysis, because of the necessity of cardioverter defibrillation therapy (Fig. 1). Baseline characteristics are listed in Table 1. T2DM patients were older than controls and well matched for BMI. Median diabetes duration was 8 years. Twenty-four of 26 T2DM patients had known CAD. Waist circumference was greater in T2DM patients than in controls. Furthermore, T2DM patients had impaired metabolic control, with higher HbA1c, triglycerides-and non-esterified fatty acid levels, and lower high-density lipoprotein (HDL) cholesterol levels. Total cholesterol and low-density lipoprotein (LDL) cholesterol levels did not differ between the groups. No difference was seen in estimated glomerular filtration rate. Yet, albumin-to-creatinine ratio was higher in T2DM patients.

**Table 1:** Baseline characteristics healthy controls and type 2 diabetic patients

	Controls (n = 10)	T2DM patients (n = 26)	P
Age, years	59 ± 5	66 ± 5	<0.01
Diabetes duration, years	NA	8 (5–11)	NA
Coronary artery disease, n (%)	NA	24 (92%)	NA
Male, n (%)	10 (100)	26 (100)	1.00
BMI, kg m^−2^	29.4 ± 2.2	29.8 ± 3.1	0.74
Waist, cm	103 ± 6	110 ± 10	0.04
Medication			
ACE inhibitor, n (%)	NA	15 (56%)	NA
Angiotensin II blocker, n (%)	NA	10 (37%)	NA
Beta blocker, n (%)	NA	23 (85%)	NA
Statins, n (%)	NA	24 (89%)	NA
Biochemical measurements (in fasten state)			
HbA1c, %	5.5 ± 0.2	7.5 ± 1.3	<0.01
Plasma glucose, mmol L^−1^	5.5 ± 0.3	9.7 ± 2.6	<0.01
Total cholesterol, mmol L^−1^	5.7 ± 0.7	4.3 ± 1.0	<0.01
HDL cholesterol, mmol L^−1^	1.4 (1.2–1.5)	1.0 (0.9–1.2)	<0.01
LDL cholesterol, mmol L	3.9 ± 0.7	2.3 ± 0.9	<0.01
Triglycerides, mmol L^−1^	1.1 (0.9–1.2)	1.6 (1.1–2.1)	0.02
Non-esterified fatty acids, mmol L^−1^	0.4 ± 0.1	0.5 ± 0.2	0.03
eGFR (MDRD), mL min^−1^ 1.73 m^−2^	89 ± 14	86 ± 28	0.80
Albumin-to-creatinin ratio, g mol^−1^	0.4 (0.3–0.5)	1.3 (0.6–2.4)	<0.01

Data are mean ± SD, median (interquartile range) or numbers of patients (percentage)

*T2DM* type 2 diabetes mellitus, *NA* not applicable, *BMI* body mass index, *ACE* angiotensin converting enzyme, *HbA1c* hemoglobin A1c, *HDL* high-density lipoprotein, *LDL* low-density lipoprotein, *eGFR* estimated glomerular filtration rate, *MDRD* modification of diet in renal disease

Hemodynamic parameters during perfusion scans are shown in Table 2. At rest, HR, SBP, and RPP were higher in T2DM patients. Hyperemia induced increased HR and RPP in both groups. In controls, hyperemia caused increased SBP and mean arterial pressure. Diastolic BP (DBP) was unaffected during hyperemia, although DBP was higher in controls than in T2DM patients during hyperemia. No differences in HR, SBP, or RPP during hyperemia between groups were observed.

**Table 2:** Hemodynamic parameters at rest and during hyperemia in healthy controls and type 2 diabetic patients

	Controls (n = 10)	T2DM patients (n = 26)	P
Heart rate, beats min^−1^			
Rest	55 ± 3	64 ± 11	<0.01
Hyperemia	76 ± 10	79 ± 13	0.42
P-value	<0.01	<0.01	
Systolic blood pressure, mmHg			
Rest	116 ± 6	127 ± 17	0.01
Hyperemia	129 ± 11	131 ± 19	0.68
P-value	<0.01	0.12	
Diastolic blood pressure, mmHg			
Rest	71 ± 6	66 ± 10	0.26
Hyperemia	74 ± 6	67 ± 8	0.02
P-value	0.21	0.47	
Mean arterial pressure, mmHg			
Rest	86 ± 5	87 ± 12	0.72
Hyperemia	92 ± 5	89 ± 11	0.17
P-value	0.02	0.23	
Rate pressure product, mm Hg min^−1^			
Rest	6318 ± 515	8050 ± 1430	<0.01
Hyperemia	9673 ± 1123	10,355 ± 2217	0.23
P-value	<0.01	<0.01	

Data are mean ± SD

*T2DM* type 2 diabetes mellitus

**Fig. 2:**
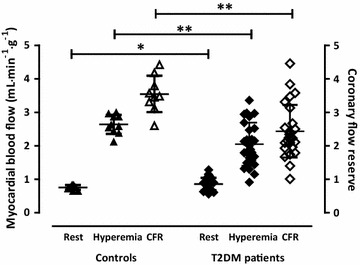
Resting, and hyperemic myocardial blood flow, and coronary flow reserve (CFR) in healthy controls (*triangles*) and type 2 diabetic (T2DM) patients (*lozenges*). *P = 0.02, ^†^P < 0.01

**Fig. 3:**
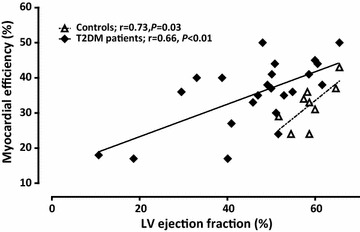
Association between left ventricular (LV) ejection fraction and myocardial efficiency in healthy controls (*white triangles*) and type 2 diabetic (T2DM) patients (*black lozenges*)

In Table 3 cardiac parameters are summarized. LV volumes and mass were not different between groups. LVEF, resting and hyperemic MBF, and CFR were impaired in T2DM patients compared to controls. T2DM patients had 6.3 g (0–11.3 g) of LV fibrosis as measured bij DCE, while controls did not show any. After correcting for RPP, differences in resting MBF and CFR were no longer present (Table 3; Fig. 2). MVO_2_ and myocardial efficiency were not different between the groups. Myocardial efficiency was related with LVEF in controls and T2DM (Fig. 3).

**Table 3:** Imaging parameters in healthy controls and type 2 diabetic patients

	Controls (n = 10)	T2DM patients (n = 26)	P
CMR			
LVEDV, mL	202 ± 36	209 ± 70	0.79
LVESV, mL	83 ± 18	117 ± 75	0.19
LV mass, g	123 ± 25	116 ± 27	0.51
LVEF, %	59 ± 4	47 ± 13	<0.01
DCE, g	0	6.3 (0–11.3)	<0.01
PET			
MBF, rest, mL^−1^ min^−1^ g^−1^	0.75 ± 0.07	0.86 ± 0.19	0.02
MBF, rest, corrected for RPP, mL min^−1^ g^−1^	1.22 ± 0.1	1.11 ± 0.4	0.20
MBF, stress, mL min^−1^ g^−1^	2.64 ± 0.28	2.05 ± 0.65	<0.01
CFR	3.54 ± 0.54	2.43 ± 0.79	<0.01
CFR, corrected for RPP, mmHg min^−1^	2.20 ± 0.35	1.97 ± 0.74	0.56
External work, Joule	1.26 ± 0.26	1.00 ± 0.31	0.04
MVO_2_, mL g^−1^ min^−1^	0.09 ± 0.01	0.08 ± 0.01	0.23
Myocardial efficiency, %	32 ± 6	35 ± 9	0.39

Data are mean ± SD or median (interquartile range)

*T2DM* type 2 diabetes mellitus, *CMR* cardiac magnetic resonance, *LVEDV* left ventricular enddiastolic volume, *LVESV* left ventricular endsystolic volume, *LVEF* left ventricular ejection fraction, *DCE* delayed contrast enhancement, *PET* positron emission tomography, *MBF* myocardial blood flow, *RPP* rate pressure product, *CFR* coronary flow reserve, *MVO*_*2*_ myocardial oxygen consumption

### Effects of exenatide and insulin glargine in T2DM patients

Eleven patients on exenatide and 12 patients on insulin glargine completed the trial (Fig. 1). Three of 14 patients randomized to exenatide discontinued after randomization, because of gastrointestinal side effects, severe LV dysfunction requiring cardiac resynchronization therapy, and one patient died from endocarditis. At baseline, the groups were well matched (Tables 4, 5). Compared to insulin glargine, exenatide reduced weight, indicated by a reduced BMI, and waist circumference (Table 4). After 26 weeks of both treatments, HbA1c decreased without between-group differences (Table 4). At follow-up, only patients on insulin glargine had decreased fasting plasma glucose (Table 4). Total cholesterol and triglycerides levels decreased after 26 weeks of exenatide, but between-group analyses showed no differences. Both HDL and LDL cholesterol, as well as non-esterified fatty acid levels were unchanged in both groups at follow-up (Table 4). Renal function, depicted in estimated glomerular filtration rate and albumin-to-creatinine ratio, was unaffected after both treatments (Table 4).

**Table 4:** 26-week treatment of exenatide versus insulin glargine, anthropometric-and biochemical parameters

	Exenatide (n = 11)			Insulin glargine (n = 12)			P between groups	
	Baseline	Follow-up	P	Baseline	Follow-up	P	Baseline	Follow-up
BMI, kg m^−2^	29.0 ± 2.6	27.6 ± 3.1	<0.01	29.9 ± 3.3	30.0 ± 3.6	0.80	0.47	<0.01
Waist, cm	108 ± 9	104 ± 10.3	<0.01	109 ± 10	110 ± 10	0.53	0.79	<0.01
Biochemical measurements (in fasten state)								
HbA1c, %	7.7 ± 1.7	7.1 ± 1.9	<0.01	7.5 ± 0.8	6.8 ± 0.7	<0.01	0.68	0.49
Plasma glucose, mmol L^−1^	10.4 ± 3.7	9.5 ± 4.0	0.14	9.3 ± 1.6	7.1 ± 1.2	<0.01	0.36	0.03
Total cholesterol, mmol L^−1^	4.1 ± 1.2	3.8 ± 0.9	0.04	4.3 ± 0.9	4.1 ± 0.9	0.06	0.74	0.25
HDL cholesterol, mmol L^−1^	0.9 (0.7–1.2)	0.9 (0.8–1.2)	0.92	1.0 (0.9–1.1)	1.0 (1.0–1.1)	0.29	0.54	0.24
LDL cholesterol, mmol L^−1^	1.9 ± 1.1	1.8 ± 0.9	0.22	2.5 ± 0.7	2.4 ± 0.8	0.17	0.17	0.65
Triglycerides, mmol L^−1^	1.8 (1.0–3.0)	1.2 (0.9–1.7)	0.04	1.8 (1.1–2.1)	1.4 (0.9–1.6)	0.11	0.87	0.33
Non-esterified fatty acids, mmol L^−1^	0.6 ± 0.1	0.5 ± 0.2	0.27	0.5 ± 0.1	0.4 ± 0.1	0.09	0.09	0.48
eGRF (MDRD), mL min^−1^ 1.73 m^−2^	90 ± 35	89 ± 26	0.65	82 ± 22	86 ± 26	0.29	0.52	0.37
Albumin-to-creatinin ratio, g mol^−1^	0.7 (0.4–1.3)	0.6 (0.4–0.7)	0.88	1.5 (0.7–3.3)	1.2 (0.7–3.5)	0.14	0.09	0.31

Data are mean ± SD, median (interquartile range) or numbers of patients (percentage)

*BMI* body mass index, *HbA1c* hemoglobin A1c, *HDL* high-density lipoprotein, *LDL* low-density lipoprotein, *eGFR* estimated glomerular filtration rate, *MDRD* modification of diet in renal disease

**Table 5:** Effects of 26-week treatment of exenatide versus insulin glargine on imaging parameters

	Exenatide (n = 11)			Insulin glargine (n = 12)			P between groups	
	Baseline	Follow-up	P	Baseline	Follow-up	P	Baseline	Follow-up
CMR								
LVEDV, mL	176 ± 42	175 ± 31	0.90	206 ± 38	206 ± 44	0.93	0.10	0.49
LVESV, mL	86 ± 26	86 ± 25	1.00	104 ± 29	109 ± 31	0.11	0.13	0.25
LV mass, g	110 ± 29	105 ± 20	0.31	113 ± 23	118 ± 23	0.27	0.82	0.05
LVEF, %	51 ± 7	52 ± 7	0.85	50 ± 9	47 ± 10	0.06	0.58	0.11
DCE, g	9.5 (1.0–11.4)	8.8 (2.2–14.0)	0.74	10.5 (5.8–21.7)	14.1 (5.3–27.4)	0.07	0.33	0.17
PET								
MBF, rest, mL min^−1^ g^−1^	0.92 ± 0.18	0.86 ± 0.11	0.33	0.80 ± 0.18	0.86 ± 0.17	0.31	0.15	0.57
MBF, rest, corrected for RPP, mL min^−1^ g^−1^	1.25 ± 0.46	1.03 ± 0.26	0.04	1.02 ± 0.26	0.98 ± 0.16	0.59	0.16	0.45
MBF, stress, mL min^−1^ g^−1^	2.29 ± 0.53	2.20 ± 0.59	0.57	2.00 ± 0.68	1.94 ± 0.62	0.46	0.28	0.79
CFR	2.59 ± 0.82	2.60 ± 0.89	0.96	2.51 ± 0.72	2.24 ± 0.59	0.20	0.82	0.22
CFR, corrected for RPP, mmHg min^−1^	2.03 ± 0.76	2.26 ± 0.79	0.19	2.04 ± 0.77	1.98 ± 0.62	0.65	0.96	0.16
External work, Joule	1.06 ± 0.38	0.98 ± 0.20	0.35	1.12 ± 0.34	1.12 ± 0.34	0.99	0.70	0.21
MVO_2_, mL g^−1^ min^−1^	0.08 ± 0.01	0.08 ± 0.01	0.52	0.08 ± 0.01	0.07 ± 0.02	0.23	0.28	0.48
Myocardial efficiency, %	36 ± 8	38 ± 6	0.53	39 ± 8	40 ± 11	0.59	0.45	0.84

Data are mean ± SD or median (interquartile range). *CMR* cardiac magnetic resonance

*LVEDV* left ventricular enddiastolic volume, *LVESV* left ventricular endsystolic volume, *LVEF* left ventricular ejection fraction, *DCE* delayed contrast enhancement, *PET* positron emission tomography, *MBF* myocardial blood flow, *RPP* rate pressure product, *CFR* coronary flow reserve, *MVO2* myocardial oxygen consumption

At follow-up, HR was increased in both groups. SBP and DBP, and mean arterial pressure, at rest and during hyperemia, were unaffected after both treatments. At follow-up, although resting RPP was increased in the exenatide group (P = 0.02), between-group analysis did not show differences for any hemodynamic parameters (not shown).

**Fig. 4:**
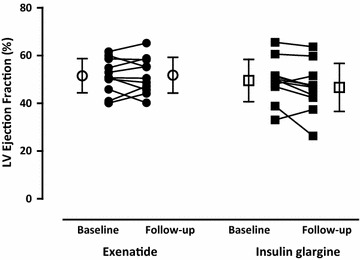
Left ventricular (LV) ejection fraction in type 2 diabetic patients at baseline and after 26-weeks of exenatide [*dots*; *open dots* (mean ± SD)] versus insulin glargine [*squares*; *open squares* (mean ± SD)]

**Fig. 5:**
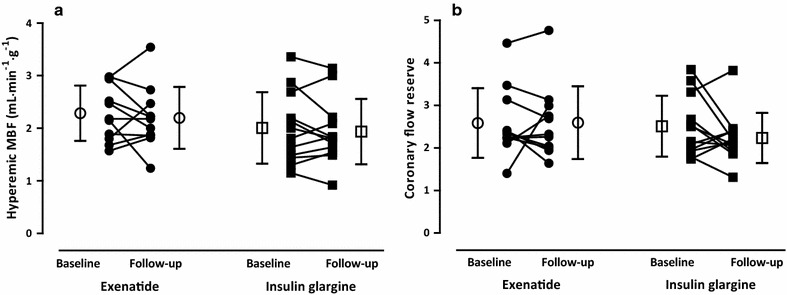
**a** Hyperemic myocardial blood flow (MBF) and **b** coronary flow reserve in type 2 diabetic patients at baseline and after 26-weeks of exenatide [*dots*; *open dots* (mean ± SD)] versus insulin glargine [*squares*; *open squares* (mean ± SD)]

**Fig. 6:**
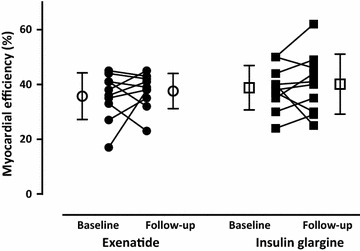
Myocardial efficiency in type 2 diabetic patients at baseline and after 26-weeks of exenatide [*dots*; *open dots* (mean ± SD)] versus insulin glargine [*squares*; *open squares* (mean ± SD)]

LV volumes and mass did not change after both treatments (Table 5). Neither LVEF, nor total DCE area, were altered at follow-up (Table 5; Fig. 4). No differences in resting (Table 5) or hyperemic MBF, as well as CFR (Table 5; Fig. 5a, c), were seen after exenatide or insulin glargine. However, RPP corrected resting MBF was decreased after exenatide, although between-group analysis did not show changes (Table 5; Fig. 5a, b). MVO_2_, and myocardial efficiency, were unchanged after both treatments (Table 5; Fig. 6).

## Discussion

The purpose of this study was to investigate the effects of exenatide compared to insulin glargine on myocardial function, perfusion, and energetics in T2DM patients with systolic dysfunction, using a BMI-matched controls as reference. As expected, T2DM patients had impaired metabolic control, with higher HbA1c, as well as fasting glucose, and non-esterified fatty acid levels, compared to controls. In addition, T2DM was associated with coronary vasomotor dysfunction, whereas myocardial energetics did not differ between groups. Systemic metabolic control improved to the same degree after 26 weeks of exenatide and insulin glargine. Finally, significant weight loss was seen after exenatide. Nevertheless, myocardial function, perfusion, and energetics were unaffected by both therapies.

### Myocardial perfusion and energetics in T2DM patients versus controls

Resting MBF was increased in T2DM as compared with controls. After correcting for hemodynamics, however, these differences were no longer apparent. Cardiovascular autonomic dysfunction is common in T2DM [23], resulting in elevated resting HR. Furthermore, as central obesity and insulin resistance are more prevalent, T2DM is associated with hypertension [24]. These changes will lead to augmentation of myocardial metabolic demand and, consequently, affect resting MBF. Existing PET data on resting MBF in T2DM are inconsistent. Without correction for hemodynamics, resting MBF in T2DM has been described as comparable to controls [25–28]. RPPs in these studies were either increased [26, 28], or comparable between T2DM patients and controls [27]. Not surprisingly, hyperemic MBF was decreased in T2DM patients, which almost all had CAD, compared to controls. However, in T2DM patients without CAD, impaired hyperemic MBF is a common finding as well [29]. As already mentioned, cardiovascular autonomic neuropathy could play a role in impairing vasodilator reserve [23, 30]. In T1DM and T2DM patients, cardiac autonomic neuropathy, assessed using [^11^C]hydroxyephedrine imaging, was associated with impaired vasodilator response, resulting in impaired hyperemic MBF as compared to diabetic patients without cardiac autonomic dysfunction [25].

Although all patients had LV systolic dysfunction, MVO_2_ and myocardial efficiency were comparable to controls. Myocardium relies predominantly on glucose and fatty acids for substrate metabolism [31]. In both T2DM [32] and HF [33], myocardial insulin resistance will lead to a shift in substrate metabolism, resulting in increased fatty acid oxidation, whilst glucose metabolism decreases. Shifting towards fatty acid metabolism is related to an (maximum) increase of 11% in oxygen consumption to generate the same amount of ATP as for glucose metabolism, resulting in impaired mechanical efficiency [34]. Further deterioration of cardiac function could be the consequence [11, 20]. There are only a few, conflicting, reports on the use of [^11^C]acetate PET to (indirectly) measure myocardial oxidative metabolism in T2DM patients. In T2DM patients without CAD, diminished myocardial efficiency and efficiency reserve were observed [35]. Peterson et al. [28] found no differences in MVO_2_ between T2DM patients and BMI-matched controls, although increased fatty acid oxidation was seen in T2DM. Rijzewijk et al. [26] reported increased fatty acid metabolism and decreased myocardial glucose uptake in patients with uncomplicated T2DM compared to healthy controls. Cardiac substrate metabolism was unrelated to LV function. In this study, all patients had LV systolic dysfunction. Idiopathic dilated cardiomyopathy is characterized by decreased myocardial efficiency and LVEF is inversely associated with myocardial fatty acid uptake [36]. Next, in patients with a history of myocardial infarction, myocardial oxygen consumption in residual viable tissues was not changed compared to CAD patients without history of myocardial infarction and controls [37]. However LV work was reduced, resulting in reduced myocardial efficiency [37]. These data are in contrast with the present findings, which did not show altered myocardial energetics in T2DM patients with predominantly ischemic cardiomyopathy.

### Effects of exenatide on myocardial function, perfusion, and energetics in T2DM patients

Hemodynamic parameters did not change after treatment with exenatide or insulin glargine. The majority of trials on GLP-1RA reported decreased SBP [38–40], LDL-cholesterol, and triglycerides [39], as well as improvements in cardiovascular risk markers as HDL-cholesterol and hsCRP [40]. These improvements sustained after 5-year of follow-up [41]. Although, increased HR was observed after 6 and 48 h of GLP-1 infusion in HF patients (with and without T2DM) [42, 43] and in a meta-analysis of 32 trials comparing GLP-1RA with placebo or active comparators, although mean HR increase was less than 2 bpm [38].

To the best of our knowledge, this is one of the first studies in which cardiac effects of long-term GLP-1RA treatment have been investigated in T2DM patients with systolic dysfunction. Neither exenatide nor insulin glargine had effects on LV dimensions or function. In contrast, initial studies investigating effects of GLP-1 in HF patients showed beneficial effects on LV function [7, 8]. In addition, 5 weeks of GLP-1 infusion improved performance in HF patients, illustrated by increased maximal O_2_-uptake and 6-min walking distance [7]. Besides, the LEADER trial [6] showed lower incidence of major cardiovascular events and cardiovascular mortality in T2DM patients with high cardiovascular risk in patients treated with liraglutide during a median follow up of 3.8 years as compared to placebo. In line with the present results, Halbirk et al. [43] could not detect an impact of GLP-1 in 20 patients without diabetes and severe systolic HF who received 48 h GLP-1 infusion. Furthermore, intervention with liraglutide during 24 weeks did not changed LV systolic function as compared with placebo in patients with chronic heart failure with and without T2DM [44]. Moreover, liraglutide administered for 180 days, did not lead to decreased numbers of rehospitalizations, and mortality, as well as N-terminal pro-B-type natriuretic peptide levels in patients recently hospitalized with heart failure and reduced LVEF [45].

Twenty-six weeks of exenatide did not affect resting or hyperemic MBF. After correction for RPP, however, resting MBF was decreased at follow-up. Clinical studies examining effects of GLP-1(RA) on coronary vascular function are scarce. In 8 T2DM patients without cardiovascular disease, resting MBF, measured using [^13^N]NH_3_ PET, increased after exenatide during a pancreatic-pituitary clamp as compared to placebo [46]. However, 10 weeks of GLP-1RA treatment caused a borderline significant increase in CFR of the left anterior descending artery in T2DM patients without CAD [47]. Others showed increased flow mediated vasodilation after either GLP-1 infusion [9] or 26 weeks of exenatide treatment [48] in T2DM. Furthermore, exenatide inhibited postprandial peripheral vascular endothelial dysfunction after a meal tolerance test, suggesting that it could have a multiphasic anti-atherogenic action involving both glucose and lipid metabolism [49]. Together, these studies and the present data suggest that GLP-1RA has little to no effect on coronary vasomotor function, and small changes in flow may actually be related to hemodynamic alterations. Moreover, most patients in this study had only modest LV dysfunction. This could have contributed to the small differences seen in cardiac function, perfusion and metabolism.

Enhancing myocardial glucose metabolism, thereby improving myocardial efficiency, has been proposed as a therapeutic strategy in HF [12]. However, in the present study MVO_2_ and myocardial efficiency were unaffected by exenatide. Reports on chronic administration of GLP-1 infusion in dogs [50] with dilated cardiomyopathy, showed increased myocardial glucose uptake. In healthy men, GLP-1 infusion did not affect myocardial glucose uptake [51]. Comparable results with exenatide infusion were shown in T2DM patients without CAD [46]. Furthermore, pre-treatment with GLP-1 in patients with coronary artery disease undergoing elective percutaneous coronary intervention, protected the heart against ischemic LV dysfunction, independent of cardiac substrate use [52]. No changes were seen in the transmyocardial glucose concentration gradients between patients randomized to either GLP-1 or placebo [52]. No changes in myocardial glucose use, oxygen consumption and myocardial efficiency were seen after albiglutide in non-diabetic patients with heart failure [53]. None of these studies, however, examined the impact on actual oxidative metabolism.

In contrary to the proposed therapeutic strategy in HF, enhancing myocardial glucose metabolism, it has been hypothesized that insulin resistance could protect the heart from substrate overload in the diabetic heart by decreasing the excess energy as myocytes are unable to convert this excess energy into mechanical energy [54]. Agents that can lower serum levels of substrates, thereby reversing the substrate overload, are therefore expected to reverse the metabolic and contractile dysfunction in the diabetic heart [54].

## Limitations

The limited sample size may have obscured potential cardiac effects of exenatide. Furthermore, 2 T2DM patients had severe LV systolic dysfunction. It is indeed well known that diastolic dysfunction frequently occurs in patients with diabetes (and obesity in general). Unfortunately, specific diastolic function parameters were not included in the current study protocol. Besides, CAD may have additionally influenced CFR, as obstructive coronary lesions were not excluded in the present study. However, these effects are likely to be small as this randomized pilot trial did not indicate any significant alterations on the investigated parameters. Furthermore, participants were all male. Therefore, these results cannot be extrapolated to female T2DM patients without further studies. Although, metabolic effects of exenatide are likely to be independent of sex [55]. To evaluate patient’s compliance all empty exenatide- and insulin glargine pens were collected. However, serum levels of exenatide were not measured. Furthermore, this study was conducted in patients with mild LV systolic dysfunction. In general, more advanced stages of HF are accompanied by cardiac device therapy prohibiting CMR imaging. Earlier data suggest that myocardial metabolism is particularly abnormal in severe HF, therefore GLP-1RA effects could be more pronounced in those patients.

## Conclusions

In T2DM patients with modest systolic dysfunction, myocardial efficiency was not impaired compared to BMI-matched healthy controls. Furthermore, 26 weeks of exenatide or insulin glargine did not result in changes in cardiac function, MBF, or oxidative metabolism.

## Abbreviations

BMI: body mass index
BP: blood pressure
CAD: coronary artery disease
CFR: coronary flow reserve
CMR: cardiovascular magnetic resonance imaging
CT: computed tomography
DCE: delayed contrast enhancement
GLP-1: glucagon like peptide-1
GLP-1RA: glucagon like peptide-1 receptor agonist
HbA1c: hemoglobin A1c
HDL: high-density lipoprotein
HF: heart failure
HR: heart rate
k_2_: rate constant for tracer washout from myocardial tissue
LDL: low-density lipoprotein
LV: left ventricular
LVEF: left ventricular ejection fraction
MBF: myocardial blood flow
MRI: magnetic resonance imaging
MVO_2_: myocardial oxygen consumption
PET: positron emission tomography
RPP: rate pressure product
SBP: systolic blood pressure
SSFP: steady-state free precession
T2DM: type 2 diabetes mellitus
